# *catena*-Poly[[di­phenyl­tin(IV)]-di-μ-iso­thio­cyanato]: an unprecedented layered coordination polymer resulting from bridging κ^2^*N*:*S* thio­cyanato ligands

**DOI:** 10.1107/S2414314624010939

**Published:** 2024-11-19

**Authors:** Ann-Kathrin Pancratz, Anne Kamrowski, Hans Reuter

**Affiliations:** a Chemistry, Osnabrück University, Barabarstr. 7, 49069 Osnabrück, Germany

**Keywords:** crystal structure, iso­thio­cyanate, di­phenyl­tin, layer structure

## Abstract

In the title compound, di­phenyl­tin(IV) diiso­thio­cyanate, [Sn(NCS)_2_(C_6_H_5_)_2_]_*n*_ or Ph_2_Sn(NCS)_2_, comparatively long tin–nitro­gen and short tin–sulfur bonds prove that the ambidentate iso­thio­cyanate ion acts as a bridge between two neighboring, octa­hedrally coordinated tin atoms. As a result, the mol­ecules lose their individuality in favor of a layered coordination polymer that represents a new type of mol­ecular inter­actions in the structural chemistry of diorganotin(IV) dihalides/pseudohalides. The tin atom is located on a center of inversion.

## Abstract 2

Di­phenyl­tin(IV) diiso­thio­cyanate, Ph_2_Sn(NCS)_2_, exhibits a new kind of inter­molecular association with loss of mol­ecular individuality and formation of a two-dimensional layer structure of octa­hedrally coordinated tin atoms linked *via* ambidentate *κ*^2^*NS* thio­cyanate ions.

## Graphical abstract

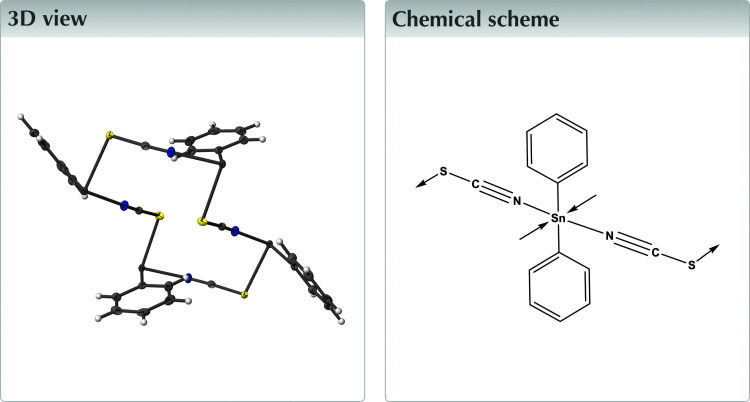

## Structure description

The thio­cyanate anion, NCS^−^, behaves like a typical pseudohalide anion consisting of a linear arrangement of three atoms and closed valence electron shells at both terminal atoms. As a mono anion, it often can replace spherical halogen atoms, whereby its dumbbell shape inevitably leads to new structural motifs. In addition, the thio­cyanate ion may act – in according with the HSAB-principle – as an ambidentate ligand that can coordinate to *hard* metal atoms *via* the small and *hard* nitro­gen atom (designation: iso­thio­cyanate) as well as to *soft* metal atoms *via* the large and *soft* sulfur atom (designation: thio­cyanate). In this context, it may coordinate to metal atoms as a monodentate (κ^1^*N*) or a bridging (κ^2^*NS*) ligand.

As the tin atom in diorganotin(IV) diiso­thio­cyanates, *R*_2_Sn(NCS)_2_, belongs to the *hard* metal atoms the NCS ligands should bind *via* the nitro­gen atoms to the tin atom in this class of compound, an assumption that was confirmed by the single-crystal structure determinations of the methyl (*R* = Me, Chow, 1970 ▸; Forder & Sheldrick, 1970 ▸; Britton, 2006 ▸) and ethyl (*R* = Et, Britton, 2006 ▸) compounds. These structure determinations reveal isolated *R*_2_Sn(NCS)_2_ mol­ecules with both *R* moieties being mutually *trans*, and small [86.09 (6)°/83.57 (4)°] angles between the thio­cyanate groups. Although the inter­molecular Sn⋯S inter­actions are weak [3.1465 (7)/3.0598 (7) Å; Britton, 2006 ▸], the mol­ecules tend to associate resulting in their chain-like arrangement with linear orientation of the dipole moments.

Here, we present the crystal structure determination of di­phenyl­tin(IV) di­thio­cyanate, Ph_2_Sn(NCS)_2_, revealing a new type of association resulting from strong Sn⋯S inter­actions and bridging thio­cyanate groups. The title compound has been known for a long time (Mullins & Curran, 1968 ▸) and has been intensively studied by IR (Mullins & Curran, 1968 ▸; Srivastava & Agarwal, 1970 ▸), NMR (Srivastava & Srivastava, 1985 ▸) and Mössbauer (Mullins & Curran, 1968 ▸) spectroscopy, especially with respect to the functionality of the thio­cyanate group and the orientation of the phenyl groups.

**Figure 1:**
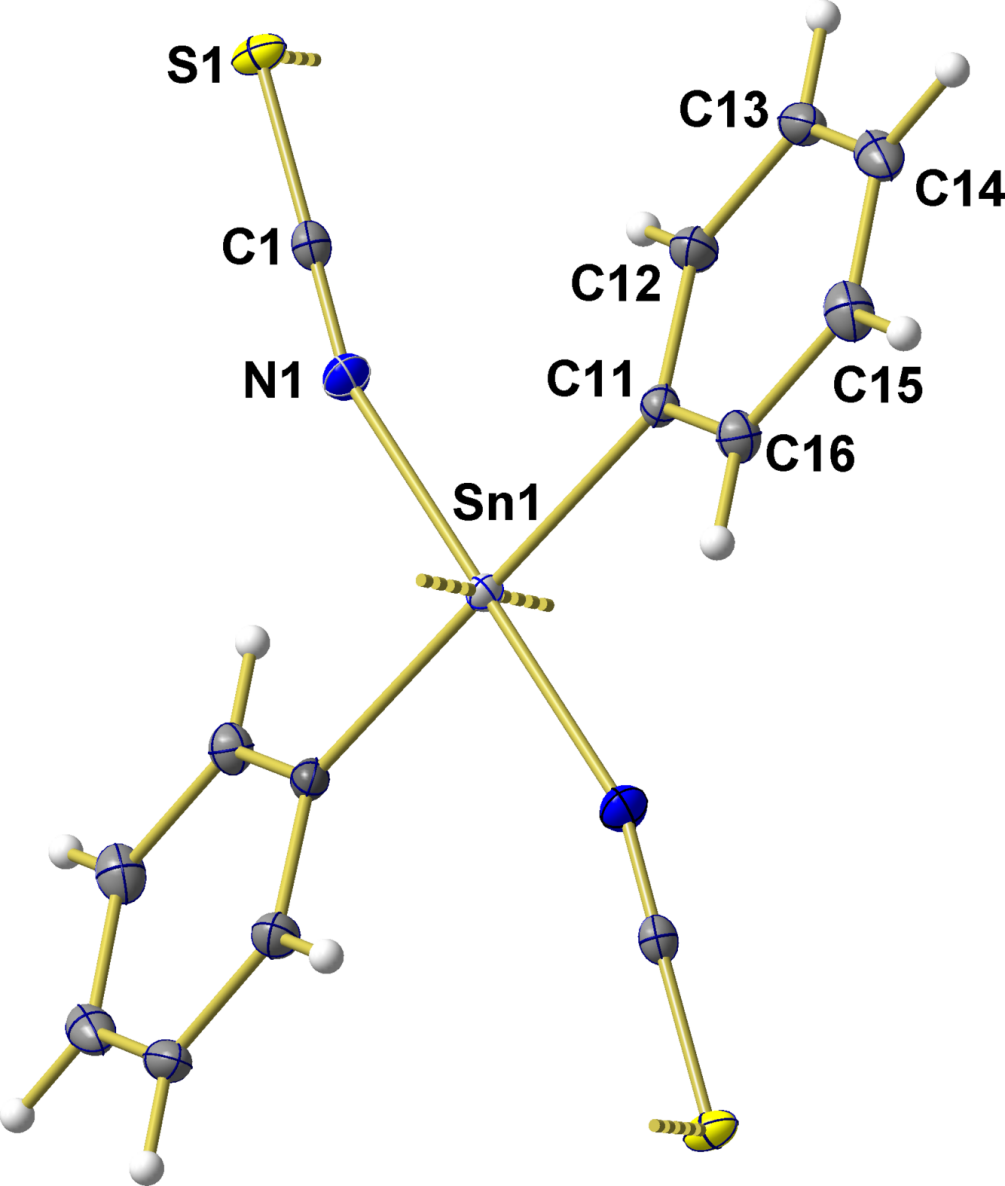
Ball-and-stick model of the centrosymmetric, octa­hedral tin environment in the crystal of Ph_2_Sn(NCS)_2_ with atom numbering given for the asymmetric unit. With exception of the hydrogen atoms, which are shown as spheres of arbitrary radius, all other atoms are drawn as displacement ellipsoids at the 40% probability level. The strong dative, sulfur–tin bonds are represented as shortened, dashed sticks.

The title compound crystallizes in the ortho­rhom­bic space group *Pbca* with four formula units in the unit cell. The asymmetric unit comprises half a formula unit with the tin atom on a center of inversion and a bridging thio­cyanato ligand, Fig. 1 ▸. In the resulting, all-*trans* configured, octa­hedral tin coordination polyhedron all dipole moments cancel each other out so that the mol­ecules lose their individuality in favor of a two-dimensional coordination polymer.

The inter­nal [*d*(C—C)_mean_ = 1.394 (7) Å, 〈(C—C—C)_mean_ = 120.0 (4)°] structural parameters of the almost [Δ_least-squares plane_ = ±0.002 (2) Å] planar phenyl group are unspectacular. As usual (Domenicano *et al.*, 1983 ▸), the inter­nal C—C—C bond angle at the *ipso* carbon atom is the smallest angle [119.4 (2)°]. The tin–carbon distance of 2.128 (4) Å compares very well with the corresponding values in the methyl [2.099 (2) Å] and ethyl [2.126 (2) Å] structures as well as with those [2.128 (5), 2.147 (6) Å] of the two crystallographic independent mol­ecules in the octa­hedral, centrosymmetric Ph_2_Sn(NCS)_2_·2(Me_2_N)_3_PO complex (Onyszchuk *et al.*, 1987 ▸) with the phenyl groups in *trans* positions. It is noteworthy that the corresponding values in the bi­pyridine complex Ph_2_Sn(NCS)_2_(bipy) (Gabe *et al.*, 1982 ▸), with the phenyl moieties in the *cis* position [〈(C—Sn—C) = 106.3°] are significantly longer [2.160 (1), 2.182 (1) Å]. A noteworthy feature in the structural chemistry of diorganotin(IV) di­thio­cyanates relates to the bond angle between the two *ipso*-carbon atoms that is exactly linear in contrast to the situation in the methy and ethyl compounds where the bond angles are 147.6 (1) and 153.0 (1)°, respectively (Britton, 2006 ▸).

**Figure 2:**
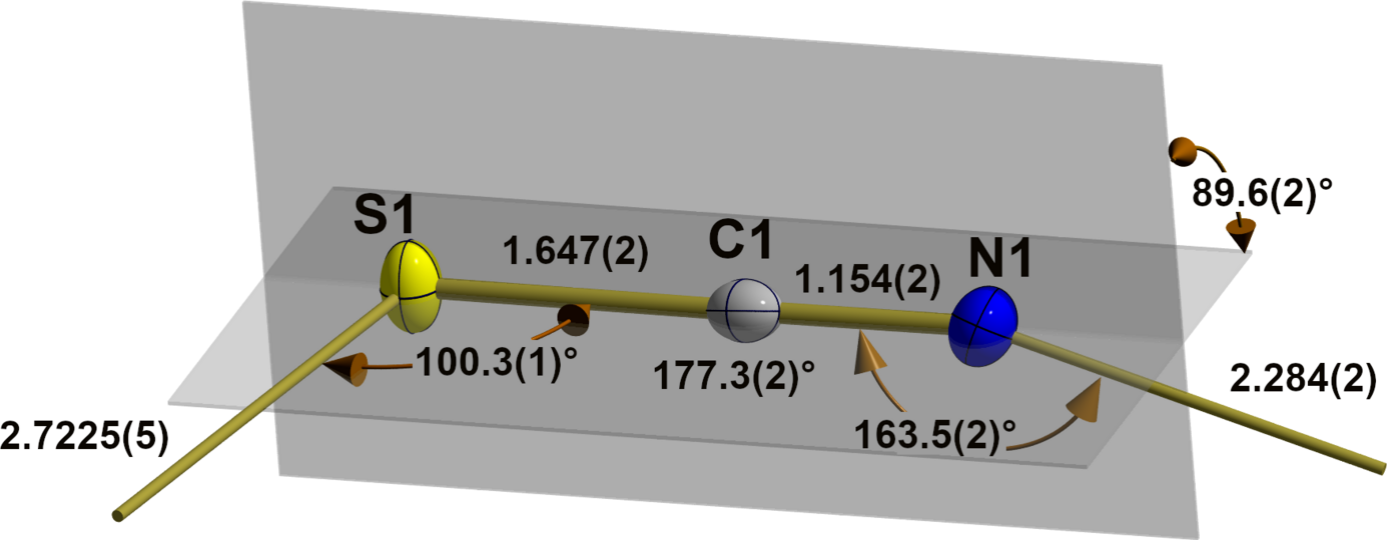
Ball-and-stick model of the thio­cyanate group in the crystal structure of Ph_2_Sn(NCS)_2_ with bond lengths (Å), bond and dihedral angles (°). All atoms are drawn as displacement ellipsoids at the 40% probability level. Nitro­gen–tin and sulfur–tin bonds are indicated by shortened sticks, planes defining the dihedral angle are shown in gray.

Regarding the thio­cyanate group (Fig. 2 ▸), the statements on its linearity, extensive rigidity of intra­molecular bond lengths, and bonding preferences were described in the recent review article on *Inorganic Metal Thio­cyanates* (Cliffe, 2024 ▸) and can be accepted without reservation (Table 1 ▸): the deviation from linearity [177.3 (2)°] is indeed more expressed than in the methyl [179.5 (2)°] and ethyl compound [179.8 (2)°] (Britton, 2006 ▸). On the other hand, the carbon–nitro­gen bond is experimentally equivalent [1.154 (2) Å] but the carbon–sulfur bond [1.647 (2)] significantly longer than in the methyl [1.159 (2)/1.615 (2) Å] and ethyl structures [1.158 (2)/1.619 (2) Å] (Britton, 2006 ▸). These values correspond very well with a carbon–nitro­gen triple [*d*(C_*sp*_≡N) = 1.155 (12) Å (Allen *et al.*, 1987 ▸)] and a carbon–sulfur single [*d*(C_*sp*_—S) = 1.630 (14) Å (Allen *et al.*, 1987 ▸)] bond.

**Figure 3:**
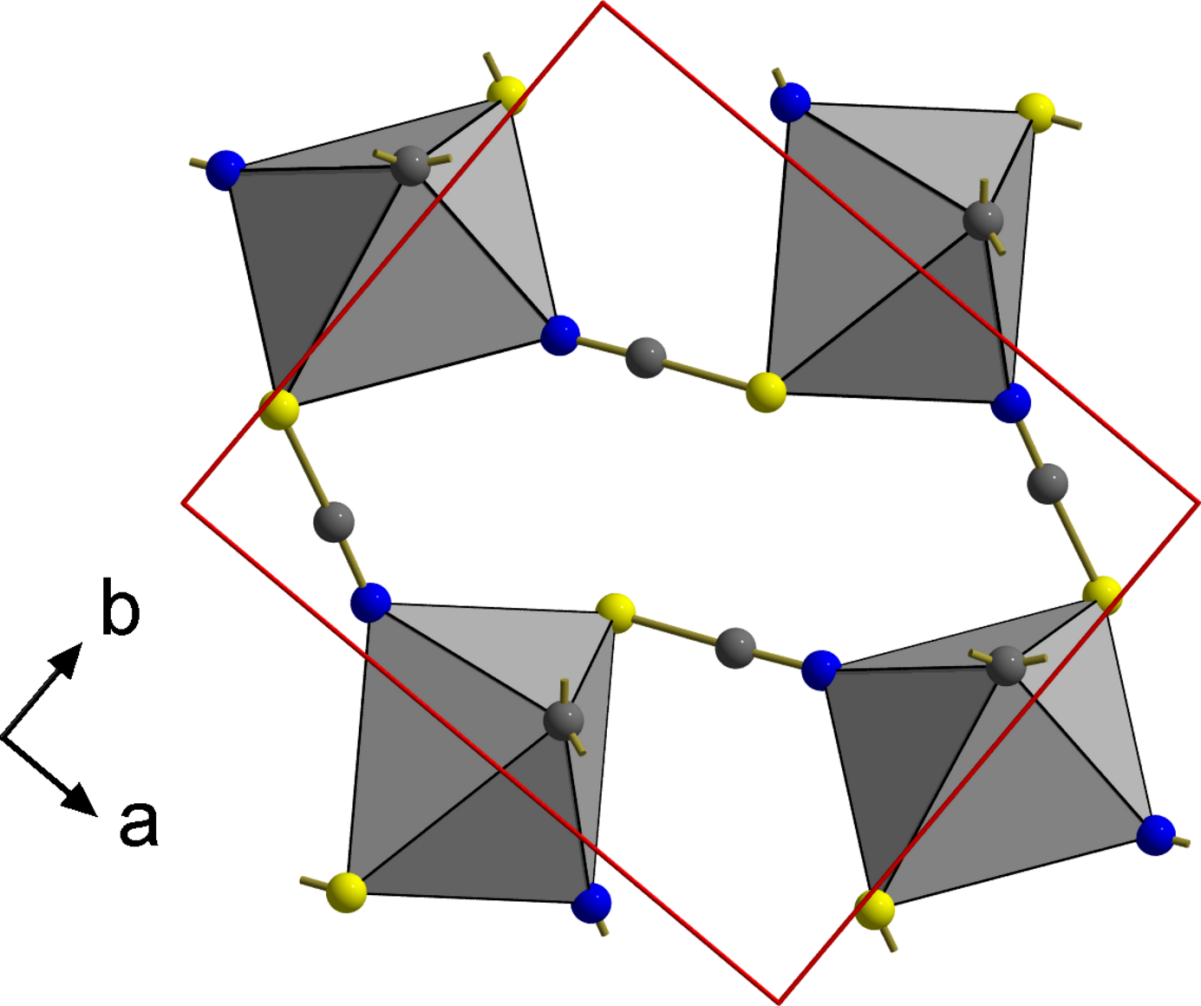
Combined ball-and-stick and polyhedron model visualizing the spacer function of the thio­cyanate groups in the layers of Ph_2_Sn(NCS)_2_ in detail. For the sake of clarity, the phenyl groups are only indicated by the *ipso*-carbon atoms and their bonds. Unit cell in red, atom color codes: S = yellow, N = blue, carbon = dark gray.

**Figure 4:**
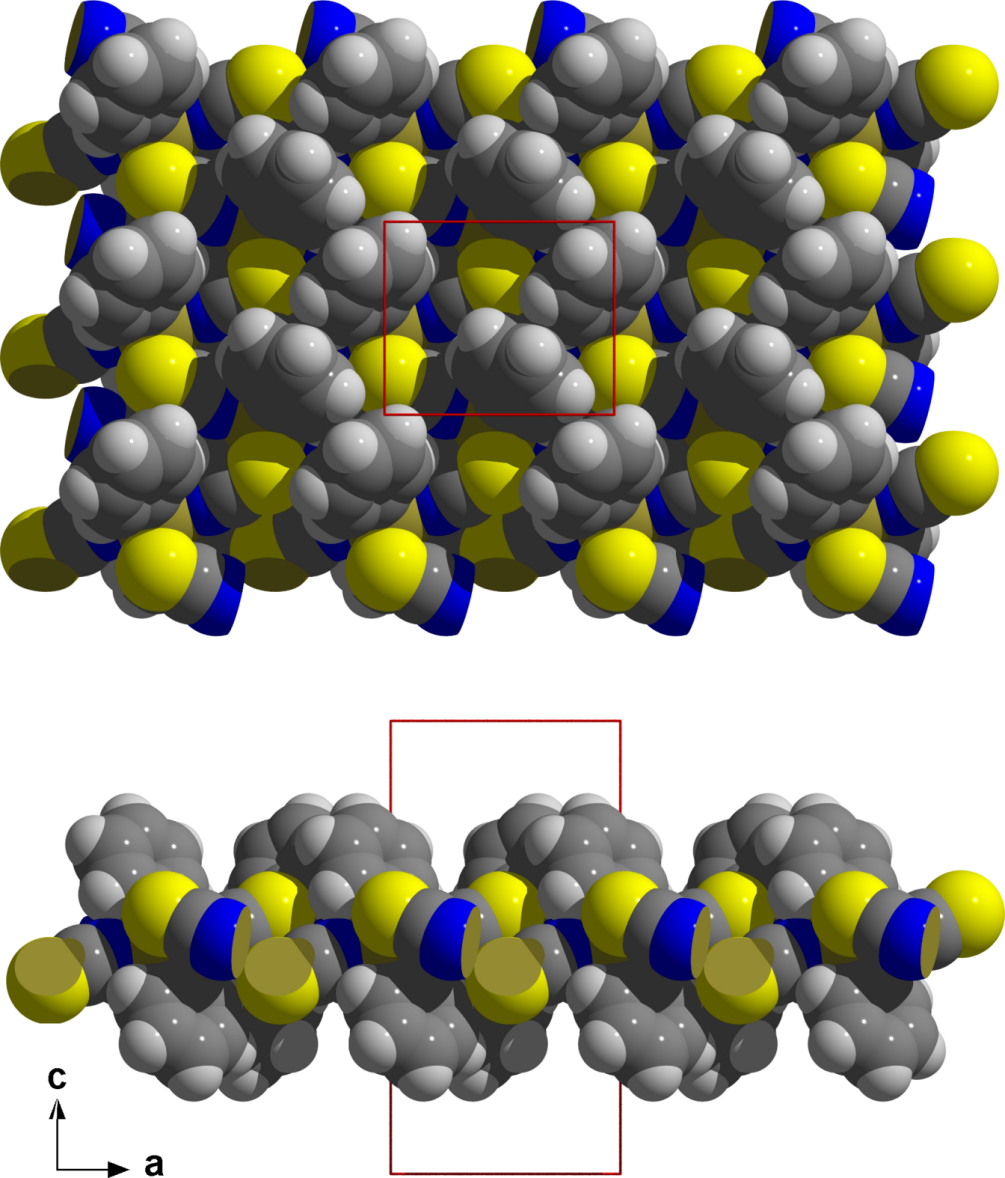
Space-filling model (top view looking down the *c* axis = above, side view looking down the *b* axis = below) of the layers in Ph_2_Sn(NCS)_2_. Atom color codes: S = yellow, N = blue, C = dark gray, H = white, tin = bronze; unit cell in red.

The extent of inter­actions with the tin atom is unique: the tin–nitro­gen bonds [*d*(Sn—N) = 2.284 (2) Å] are considerable longer [+0.155/+0.132 Å] and the tin-sulfur inter­actions [*d*(Sn—S) = 2.7224 (5) Å] significant shorter [−0.425/−0.338 Å] than in the methyl and ethyl structures (Britton, 2006 ▸). Since the tin atom lies on a center of symmetry, it is surrounded by two sulfur atoms that are exactly *trans* to each other while they are *cis* with bonding angles of 86.09 (6)° [*R* = Me] and 83.57 (4)° [*R* = Et]. The orientation of the bridging thio­cyanate ion in the coordination sphere of the tin atom is characterized by a Sn—N—C bond angle of 163.45 (15)° [164.2 (1)°/164.5 (1)°, Me/Et] and a Sn—S—C bond angle of 100.31 (6)° [91.83 (6)°/91.92 (6)°, Me/Et], while the torsion angle Sn—N(C)S—Sn′ amounts to 89.6 (2)°, but 0° and 15.9 (2)°, respectively, in the methyl and ethyl structures. In summary, this strong bridging function of the thio­cyanate ions leads to a layer structure of {Sn*R*_2_N_2_S_2_}-octa­hedra corner-linked *via* the thio­cyanate groups as spacers (Fig. 3 ▸). Their orientation in relation to the plane of the tin atoms is given by a distance of ±0.331 (2) Å of the nitro­gen and ±1.3679 (4) Å of the sulfur atom. The angle between the plane and the NCS-dumbbells is 22.74 (4)° (Fig. 4 ▸). The layers are stacked in the direction of the *c* axis in such a way that the tips of the phenyl residues of one layer fall into the bulges of the other.

In the structure chemistry of the diorganotin(IV) dihalides, *R*_2_SnHal_2_, a similar type of (001) layer structure is only known from di­methyl­tin(IV) difluoride, Me_2_SnF_2_, for which a tetra­gonal unit cell is reported in the literature (Schlemper & Hamilton, 1966 ▸), in which {Me_2_SnF_4/2_} octa­hedra are linked *via* the fluorine atoms to planar Sn—F layers.

## Synthesis and crystallization

For the synthesis from sodium thio­cyanate and di­phenyl­tin(IV) dichloride in ethanol (mole ratio 1:2), elemental analysis, and melting point see Mullins & Curran (1968 ▸), Srivastava & Agarwal (1970 ▸). Colorless, plate-like single crystals were obtained by recrystallization from ethanol.

## Refinement

Crystal data, data collection and structure refinement details are summarized in Table 2 ▸.

## Supplementary Material

Crystal structure: contains datablock(s) I. DOI: 10.1107/S2414314624010939/tk4112sup1.cif

Structure factors: contains datablock(s) I. DOI: 10.1107/S2414314624010939/tk4112Isup2.hkl

CCDC references: 2402079, 2402079

Additional supporting information:  crystallographic information; 3D view; checkCIF report

## Figures and Tables

**Table 1:** Selected geometric parameters (Å, °)

Sn1—N1	2.284 (2)	N1—C1	1.154 (2)
Sn1—S1^i^	2.7224 (5)	C1—S1	1.647 (2)
			
C1—N1—Sn1	163.5 (2)	N1—C1—S1	177.3 (2)
C1—S1—Sn1^ii^	100.31 (6)		

Symmetry codes: (i) 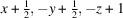; (ii) 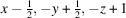.

**Table 2:** Experimental details

Crystal data	
Chemical formula	[Sn(NCS)_2_(C_6_H_5_)_2_]
*M*_r_	389.05
Crystal system, space group	Orthorhombic, *Pbca*
Temperature (K)	100
*a*, *b*, *c* (Å)	9.4438 (4), 7.9296 (3), 18.6153 (8)
*V* (Å^3^)	1394.02 (10)
*Z*	4
Radiation type	Mo *K*α
μ (mm^−1^)	2.12
Crystal size (mm)	0.25 × 0.14 × 0.09

Data collection	
Diffractometer	Bruker APEXII CCD
Absorption correction	Multi-scan (*SADABS*; Krause *et al.*, 2015 ▸)
*T*_min_, *T*_max_	0.454, 0.712
No. of measured, independent and observed [*I* > 2σ(*I*)] reflections	32393, 1689, 1453
*R*_int_	0.078
(sin θ/λ)_max_ (Å^−1^)	0.660

Refinement	
*R*[*F*^2^ > 2σ(*F*^2^)], *wR*(*F*^2^), *S*	0.021, 0.055, 1.08
No. of reflections	1689
No. of parameters	90
H-atom treatment	Only H-atom displacement parameters refined
Δρ_max_, Δρ_min_ (e Å^−3^)	0.42, −0.37

Computer programs: *APEX2* and *SAINT* (Bruker, 2009 ▸), *SHELXS97* (Sheldrick, 2008 ▸), *SHELXL2014/7* (Sheldrick, 2015 ▸), *DIAMOND* (Brandenburg, 2006 ▸), *Mercury* (Macrae *et al.*, 2020 ▸) and *publCIF* (Westrip, 2010 ▸).

## full crystallographic data

### catena-Poly[[diphenyltin(IV)]-di-µ-isothiocyanato] Crystal data

**Table:**

[Sn(NCS)_2_(C_6_H_5_)_2_]	*D*_x_ = 1.854 Mg m^−^^3^
*M**_r_* = 389.05	Mo *K*α radiation, λ = 0.71073 Å
Orthorhombic, *P**b**c**a*	Cell parameters from 8887 reflections
*a* = 9.4438 (4) Å	θ = 4.0–29.5°
*b* = 7.9296 (3) Å	µ = 2.12 mm^−^^1^
*c* = 18.6153 (8) Å	*T* = 100 K
*V* = 1394.02 (10) Å^3^	Plate, colourless
*Z* = 4	0.25 × 0.14 × 0.09 mm
*F*(000) = 760	

### catena-Poly[[diphenyltin(IV)]-di-µ-isothiocyanato] Data collection

**Table:**

Bruker APEXII CCD diffractometer	1453 reflections with *I* > 2σ(*I*)
φ and ω scans	*R*_int_ = 0.078
Absorption correction: multi-scan (SADABS; Krause *et al.*, 2015)	θ_max_ = 28.0°, θ_min_ = 3.1°
*T*_min_ = 0.454, *T*_max_ = 0.712	*h* = −12→12
32393 measured reflections	*k* = −10→10
1689 independent reflections	*l* = −24→24

### catena-Poly[[diphenyltin(IV)]-di-µ-isothiocyanato] Refinement

**Table:**

Refinement on *F*^2^	Hydrogen site location: inferred from neighbouring sites
Least-squares matrix: full	Only H-atom displacement parameters refined
*R*[*F*^2^ > 2σ(*F*^2^)] = 0.021	*w* = 1/[σ^2^(*F*_o_^2^) + (0.0174*P*)^2^ + 1.1927*P*] where *P* = (*F*_o_^2^ + 2*F*_c_^2^)/3
*wR*(*F*^2^) = 0.055	(Δ/σ)_max_ = 0.001
*S* = 1.08	Δρ_max_ = 0.42 e Å^−^^3^
1689 reflections	Δρ_min_ = −0.37 e Å^−^^3^
90 parameters	Extinction correction: *SHELXL2014/7* (Sheldrick, 2015), Fc^*^=kFc[1+0.001xFc^2^λ^3^/sin(2θ)]^-1/4^
0 restraints	Extinction coefficient: 0.0036 (3)
Primary atom site location: structure-invariant direct methods	

### catena-Poly[[diphenyltin(IV)]-di-µ-isothiocyanato] Special details

**Table:**

Geometry. All esds (except the esd in the dihedral angle between two l.s. planes) are estimated using the full covariance matrix. The cell esds are taken into account individually in the estimation of esds in distances, angles and torsion angles; correlations between esds in cell parameters are only used when they are defined by crystal symmetry. An approximate (isotropic) treatment of cell esds is used for estimating esds involving l.s. planes.

### catena-Poly[[diphenyltin(IV)]-di-µ-isothiocyanato] Fractional atomic coordinates and isotropic or equivalent isotropic displacement parameters (Å^2^)

**Table:**

	*x*	*y*	*z*	*U*_iso_*/*U*_eq_	
Sn1	0.5000	0.0000	0.5000	0.01193 (9)	
C11	0.55455 (19)	0.1194 (2)	0.59859 (9)	0.0141 (3)	
C12	0.4693 (2)	0.2473 (2)	0.62674 (10)	0.0196 (4)	
H12	0.3890	0.2855	0.6007	0.026 (3)*	
C13	0.5021 (2)	0.3190 (3)	0.69315 (11)	0.0224 (5)	
H13	0.4429	0.4043	0.7127	0.026 (3)*	
C14	0.6209 (2)	0.2660 (2)	0.73066 (10)	0.0231 (4)	
H14	0.6429	0.3146	0.7759	0.026 (3)*	
C15	0.7072 (2)	0.1425 (2)	0.70216 (10)	0.0215 (4)	
H15	0.7897	0.1080	0.7275	0.026 (3)*	
C16	0.6741 (2)	0.0678 (2)	0.63639 (10)	0.0178 (4)	
H16	0.7332	−0.0182	0.6174	0.026 (3)*	
N1	0.26755 (17)	0.0675 (2)	0.51778 (9)	0.0187 (3)	
C1	0.16417 (19)	0.1262 (2)	0.53937 (9)	0.0145 (3)	
S1	0.01711 (5)	0.20384 (6)	0.57348 (2)	0.01715 (12)	

### catena-Poly[[diphenyltin(IV)]-di-µ-isothiocyanato] Atomic displacement parameters (Å^2^)

**Table:**

	*U*^11^	*U*^22^	*U*^33^	*U*^12^	*U*^13^	*U*^23^
Sn1	0.00918 (14)	0.01226 (12)	0.01434 (12)	0.00020 (5)	−0.00024 (6)	−0.00147 (5)
C11	0.0133 (9)	0.0142 (7)	0.0147 (7)	−0.0028 (7)	0.0004 (7)	0.0003 (6)
C12	0.0177 (9)	0.0187 (9)	0.0223 (9)	0.0003 (8)	0.0012 (8)	−0.0037 (7)
C13	0.0251 (12)	0.0182 (9)	0.0238 (9)	−0.0031 (7)	0.0092 (8)	−0.0059 (8)
C14	0.0345 (12)	0.0208 (9)	0.0141 (8)	−0.0086 (8)	0.0036 (8)	−0.0001 (7)
C15	0.0262 (11)	0.0199 (9)	0.0184 (9)	−0.0035 (8)	−0.0052 (8)	0.0034 (7)
C16	0.0187 (10)	0.0155 (8)	0.0192 (9)	−0.0009 (7)	−0.0014 (7)	0.0005 (7)
N1	0.0128 (9)	0.0200 (8)	0.0233 (7)	0.0014 (7)	0.0003 (7)	−0.0021 (7)
C1	0.0158 (9)	0.0128 (8)	0.0150 (8)	−0.0020 (7)	−0.0028 (7)	0.0002 (6)
S1	0.0156 (2)	0.0168 (2)	0.0191 (2)	0.00447 (17)	0.00561 (17)	0.00345 (17)

### catena-Poly[[diphenyltin(IV)]-di-µ-isothiocyanato] Geometric parameters (Å, º)

**Table:**

Sn1—C11^i^	2.128 (2)	C12—C13	1.395 (3)
Sn1—C11	2.128 (2)	C13—C14	1.387 (3)
Sn1—N1^i^	2.284 (2)	C14—C15	1.380 (3)
Sn1—N1	2.284 (2)	C15—C16	1.395 (2)
Sn1—S1^ii^	2.7224 (5)	N1—C1	1.154 (2)
Sn1—S1^iii^	2.7224 (5)	C1—S1	1.647 (2)
C11—C16	1.392 (3)	S1—Sn1^iv^	2.7225 (5)
C11—C12	1.397 (3)		
			
C11^i^—Sn1—C11	180.00 (8)	N1—Sn1—S1^iii^	85.85 (4)
C11^i^—Sn1—N1^i^	90.19 (6)	S1^ii^—Sn1—S1^iii^	180.0
C11—Sn1—N1^i^	89.81 (6)	C16—C11—C12	119.4 (2)
C11^i^—Sn1—N1	89.81 (6)	C16—C11—Sn1	120.1 (1)
C11—Sn1—N1	90.19 (6)	C12—C11—Sn1	120.5 (1)
N1^i^—Sn1—N1	180.00 (2)	C13—C12—C11	120.1 (2)
C11^i^—Sn1—S1^ii^	92.02 (5)	C14—C13—C12	120.1 (2)
C11—Sn1—S1^ii^	87.98 (5)	C15—C14—C13	120.0 (2)
N1^i^—Sn1—S1^ii^	85.85 (4)	C14—C15—C16	120.4 (2)
N1—Sn1—S1^ii^	94.15 (4)	C11—C16—C15	120.1 (2)
C11^i^—Sn1—S1^iii^	87.98 (5)	C1—N1—Sn1	163.5 (2)
C11—Sn1—S1^iii^	92.02 (5)	C1—S1—Sn1^iv^	100.31 (6)
N1^i^—Sn1—S1^iii^	94.15 (4)	N1—C1—S1	177.3 (2)

Symmetry codes: (i) −*x*+1, −*y*, −*z*+1; (ii) −*x*+1/2, *y*−1/2, *z*; (iii) *x*+1/2, −*y*+1/2, −*z*+1; (iv) *x*−1/2, −*y*+1/2, −*z*+1.
